# Time-Dependent Increase in Medial Meniscus Extrusion Predicts the Need for Meniscal Repair in Patients with Partial Medial Meniscus Posterior Root Tears: A Case–Control Study

**DOI:** 10.1007/s43465-023-00987-3

**Published:** 2023-09-05

**Authors:** Koki Kawada, Takayuki Furumatsu, Masanori Tamura, Haowei Xue, Naohiro Higashihara, Keisuke Kintaka, Yusuke Yokoyama, Toshifumi Ozaki

**Affiliations:** Department of Orthopaedic Surgery, Okayama University Graduate School of Medicine, Dentistry and Pharmaceutical Sciences, 2-5-1 Shikata-cho, Kita-ku, Okayama, 700-8558 Japan

**Keywords:** Meniscus, Posterior root tear, Conservative treatment, Partial tear, Meniscus extrusion

## Abstract

**Purpose:**

This study aimed to compare medial meniscus extrusion (MME) in patients with partial medial meniscus posterior root tears (MMPRTs) through magnetic resonance imaging (MRI) conducted at two-time points and to determine whether patient characteristics or MME measurements differ in patients who respond to nonoperative treatment compared with those who require surgical treatment.

**Methods:**

Thirty-seven patients with partial MMPRTs underwent two MRI scans during nonoperative management or before pull-out repair. Among these, 17 patients received nonoperative management, and 20 underwent pull-out repair. Partial MMPRTs were diagnosed based on the MRI findings. MME measurements were performed on both MRI scans. Statistical and receiver operating curve (ROC) analyses were performed.

**Results:**

The duration between the two MRI scans was significantly shorter in the pull-out repair group than in the nonoperative management group. The increase in MME (ΔMME) on MRI scans was significantly greater in the pull-out repair group than in the nonoperative management group. Linear regression analysis revealed a weak correlation between the MRI interval and ΔMME in the nonoperative management group and a moderate correlation in the pull-out repair group. In the ROC construction, the cut-off value for ΔMME that requires surgical intervention was 0.41 mm, with a sensitivity and specificity of 85.0% and 52.9%, respectively.

**Conclusion:**

Patients with partial MMPRTs requiring surgical treatment had greater MME progression in a shorter time and a time-dependent increase in MME. Therefore, a ΔMME of ≥ 0.41 mm may be useful in deciding surgical intervention based on MRI retests.

**Level of evidence:**

III.

## Introduction

Medial meniscus posterior root tears (MMPRTs) occur frequently in middle-aged adults and cause painful posteromedial popping with descending actions, such as stairs or downhill [1]. MMPRTs disrupt the hoop function of the meniscus, increasing medial compartment load and causing cartilage damage [2, 3]. Recently, meniscal repair has been widely performed for MMPRTs and is more effective than conservative therapy or meniscectomy in preventing cartilage damage and reducing additional surgeries [4].

According to LaPrade's classification, partial MMPRTs are classified as type 1 [5]. The prevalence of partial MMPRTs based on arthroscopic findings was 7.0% (5/71 knees) [5], 16.1% (9/56 knees) [6], and 16.4% (19/116 knees) [7]. Partial MMPRTs do not always require surgical treatment. However, some patients may require posterior root repairs due to severe knee pain and associated difficulties in daily living. Moreover, partial MMPRTs may progress to complete MMPRTs before diagnosis or during conservative treatment [8]. Consequently, understanding the surgical indications for partial MMPRTs is crucial for preventing partial MMPRTs from becoming complete MMPRTs and further progression of arthropathic changes. Currently, the surgical indications for partial MMPRTs are controversial.

Therefore, this study aimed to compare medial meniscus extrusion (MME) measured using magnetic resonance imaging (MRI) performed at two-time points in patients with partial MMPRTs and determine whether patient characteristics or MME measurements differ in patients who respond to nonoperative treatment compared with those who require surgical treatment.

We hypothesized that patients requiring surgical intervention with partial MMPRTs would have rapid MME progression and a time-dependent MME increase.

## Materials and Methods

### Patient Selection Criteria

This retrospective case–control study was approved by our Institutional Review Board. Written informed consent was obtained from all patients included in the study. This study indications were tibiofemoral angle < 180°, Kellgren–Lawrence (KL) grade 0–2, and mild cartilage lesions. Surgical indications were not affected by age, height, weight, body mass index, or patient activity.

**Fig. 1:**
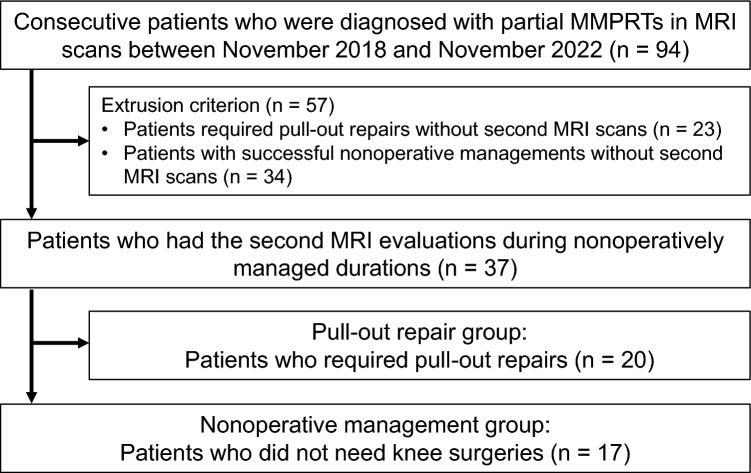
Study protocol flowchart. MMPRTs, medial meniscus posterior root tears; MRI, magnetic resonance imaging

Ninety-four consecutive patients diagnosed with partial MMPRTs by MRI between November 2018 and November 2022 were retrospectively reviewed (Fig. 1). Among them, 57 patients were excluded: 23 who required pull-out repair without a second MRI scan and 34 who received successful conservative treatment without a second MRI scan. Thirty-seven patients who underwent two MRI scans during nonoperative management or prior to pull-out repair were included in this study: 17 in the nonoperative management group and 20 in the pull-out repair group. A second MRI scan was performed for reassessment in patients with persistent or recurrent knee pain or those with knee pain more severe than assessed during the first MRI scan.

**Fig. 2:**
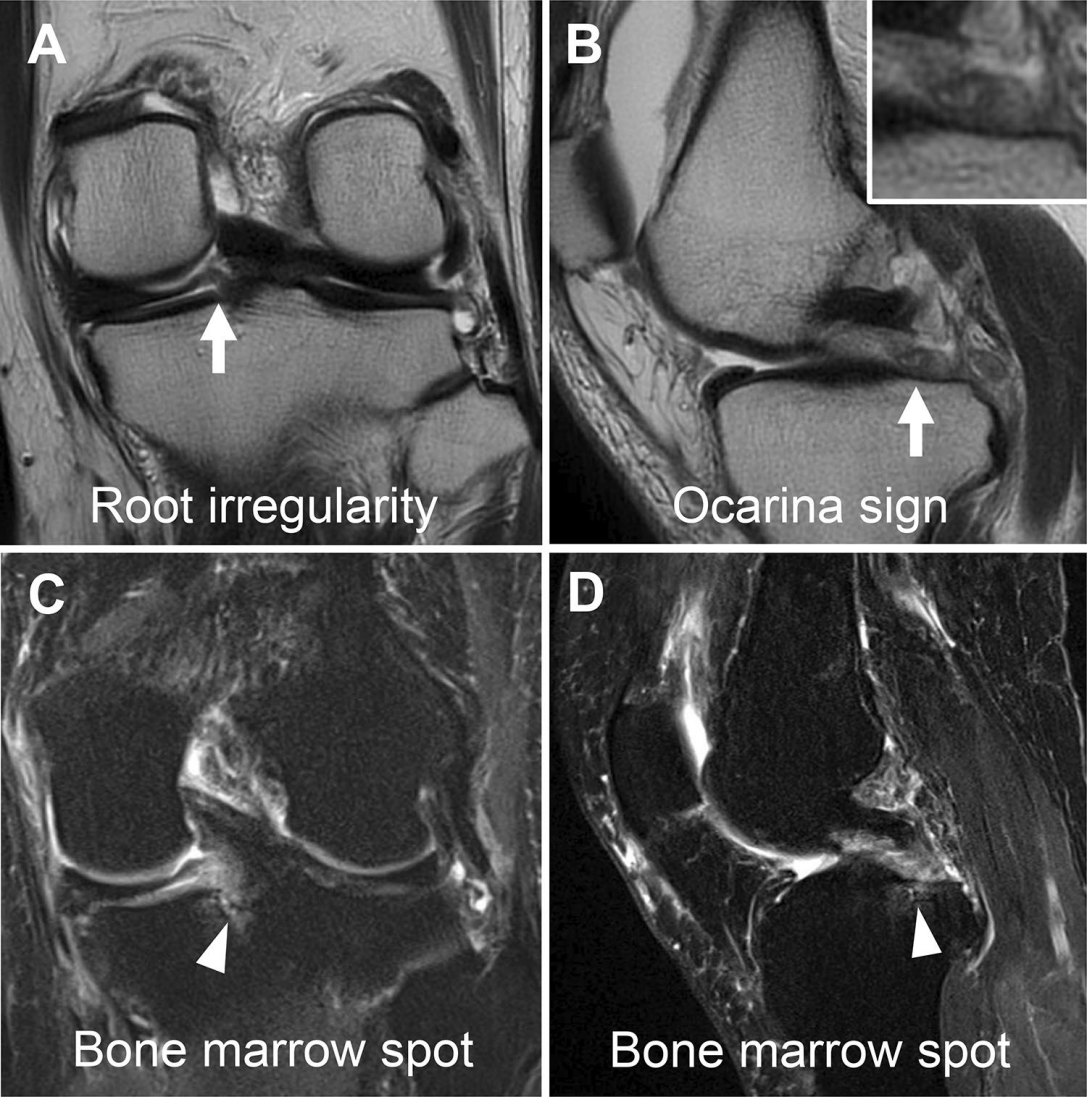
MRI findings of partial MMPRTs. Partial MMPRTs diagnosed via MRI. **a** Root irregularity (arrow, coronal T2-weighted image). **b** Ocarina sign (arrow, sagittal T2-weighted image). **c** Bone marrow spot (arrowhead, coronal T2-weighted fat-suppressed image). **d** Bone marrow spot (arrowhead, sagittal T2-weighted fat-suppressed image). *MMPRTs* medial meniscus posterior root tears, *MRI* magnetic resonance imaging

Partial MMPRTs were diagnosed based on MRI findings of the ocarina sign [6], root irregularity, and bone marrow spot [9] (Fig. 2). The ocarina sign represents multiple spotty signal changes in the posterior root region on MRI sagittal images and has been reported as a suspicious finding in the case of partial MMPRTs [6]. MRI findings, such as ghosts, giraffe neck, and cleft signs, were diagnosed as complete MMPRTs and excluded from this study.

Partial MMPRTs were confirmed under arthroscopy in all patients who underwent pull-out repair, and the onset date was determined through interviews.

### MRI-Based Measurements

All patients underwent two MRI scans during nonoperative management or prior to pull-out repair. The first MRI findings of partial MMPRTs were investigated for root irregularity, bone marrow spot, and ocarina sign. MME measurements were recorded with both MRI scans. MME was defined as the distance from the medial margin of the tibial plateau to the medial margin of the medial meniscus (MM), excluding osteophytes in the coronal slice at the anterior–posterior MM length midpoint. The MME was recorded up to the second decimal place.

### Surgical Indications

Initially, patients with partial MMPRTs were conservatively treated. Nonoperative management included daily lifestyle guidance, such as avoiding deep knee flexion, losing weight, quadriceps muscle training, and pain control with analgesics. Although some patients could not apply weight due to pain, they were not instructed to be unloaded. No clinical scale was used as a definitive criterion for determining surgical interventions, although the surgical intervention was determined through group discussion based on a comprehensive evaluation of the patient's persistent pain, difficulties in daily living, and the patient's choice. If surgical intervention was chosen, patients underwent transtibial pull-out repair.

### Statistical Analyses

Values are expressed as mean ± standard deviation. Statistical and receiver operating curve (ROC) analyses were performed using EZR (Saitama Medical Centre, Saitama, Japan). Fisher’s exact test or Mann–Whitney U test was used in comparing both groups. Statistical significance was set at *p* < 0.05. Linear regression analysis was used to evaluate the correlation between the MRI interval and an increase in MME (ΔMME). High, moderate, and low correlation was represented by R^2^ ≥ 0.6, 0.6 > R^2^ ≥ 0.4, and R^2^ < 0.4, respectively.

Two orthopaedic surgeons were blinded to the MME measurements and independently assessed them. Each observer performed each evaluation two times, at least 6 weeks apart. The inter- and intra-observer reliability of the measurements was examined using intraclass correlation coefficients (ICC).

## Results

Patient sex, age, height, body weight, and body mass index were not significantly different between the nonoperative management and pull-out repair groups (Table 1). The duration from the onset to the first MRI scan (23.2 ± 16.1 days vs. 23.9 ± 37.8 days, *p* = 0.200) and the follow-up duration (22.6 ± 12.2 months vs. 25.1 ± 12.8 months, *p* = 0.583) were not significantly different between the two groups. The duration between the two MRI scans was significantly shorter in the pull-out repair group (46.4 ± 45.8 days) than in the nonoperative management group (80.2 ± 38.0 days, *p* = 0.001).

**Table 1:** Patient characteristics

	Nonoperative	Pull-out repair	*p*-value
Number of patients	17	20	
Sex, male/female	2/15	7/13	0.136
Age (years)	62.2 ± 9.3	65.0 ± 7.6	0.552
[range]	[39–75]	[48–79]	
Height (m)	1.57 ± 0.07	1.58 ± 0.06	0.836
[range]	[1.44–1.68]	[1.49–1.72]	
Body weight (kg)	67.0 ± 24.9	64.9 ± 10.1	0.509
[range]	[43–140]	[46–88]	
Body mass index (kg/m^2^)	26.6 ± 7.7	25.8 ± 3.0	0.451
[range]	[20.7–49.6]	[19.9–33.9]	
Duration from onset to first MRI (days)	23.2 ± 16.1	23.9 ± 37.8	0.200
[range]	[1–74]	[1–169]	
Duration from first MRI to second MRI (days)	80.2 ± 38.0	46.4 ± 45.8	0.001*
[range]	[40–173]	[19–226]	
Follow-up duration (months)	22.6 ± 12.2	25.1 ± 12.8	0.583
[range]	[9–62]	[11–65]	

Values are presented as the mean ± standard deviation or number. *p*-values were derived using Fisher’s exact or Mann–Whitney U test

*MRI* magnetic resonance imaging

*Statistically significant

On the first MRI scan, the MME (2.48 ± 0.73 mm vs. 2.78 ± 0.72 mm, *p* = 0.223), MME of > 3 mm (17.6% vs. 35.0%, *p* = 0.288), and the frequency of MRI findings suggestive of partial MMPRTs did not differ significantly between the two groups (Table 2). However, on the second MRI scan, the MME (2.92 ± 0.83 mm vs. 3.56 ± 0.65 mm, *p* = 0.010) and MME of > 3 mm (41.2% vs. 75.0%, *p* = 0.050) were significantly larger in the pull-out repair group than in the nonoperative management group. Additionally, the ΔMME based on the two MRIs was significantly greater in the pull-out group (0.78 ± 0.41 mm) than in the nonoperative management group (0.44 ± 0.37 mm, *p* = 0.021).

**Table 2:** Comparison of MRI findings in nonoperative management and pull-out repair groups

	Nonoperative (n = 17)	Pull-out repair (n = 20)	*p-*value
Root irregularity (%)	6 (35.3)	10 (50.0)	0.508
Bone marrow spot (%)	9 (52.9)	9 (45.0)	0.746
Ocarina sign (%)	17 (100)	20 (100)	1.000
MME in the first MRI (mm)	2.48 ± 0.73	2.78 ± 0.72	0.223
[range]	[1.40–4.50]	[1.73–4.06]	
MME in the second MRI (mm)	2.92 ± 0.83	3.56 ± 0.65	0.010*
[range]	[1.50–5.25]	[2.56–4.48]	
ΔMME (mm)	0.44 ± 0.37	0.78 ± 0.41	0.021*
[range]	[0.00–1.20]	[0.26–1.87]	
MME > 3 mm in the first MRI (%)	3 (17.6)	7 (35.0)	0.288
MME > 3 mm in the second MRI (%)	7 (41.2)	15 (75.0)	0.050*

Values are presented as the mean ± standard deviation. *p*-values were derived using Fisher’s exact test or Mann–Whitney U test

*MME* medial meniscus extrusion, *MRI* magnetic resonance imaging, *ΔMME* the increase in medial meniscus extrusion

*Statistically significant

**Fig. 3:**
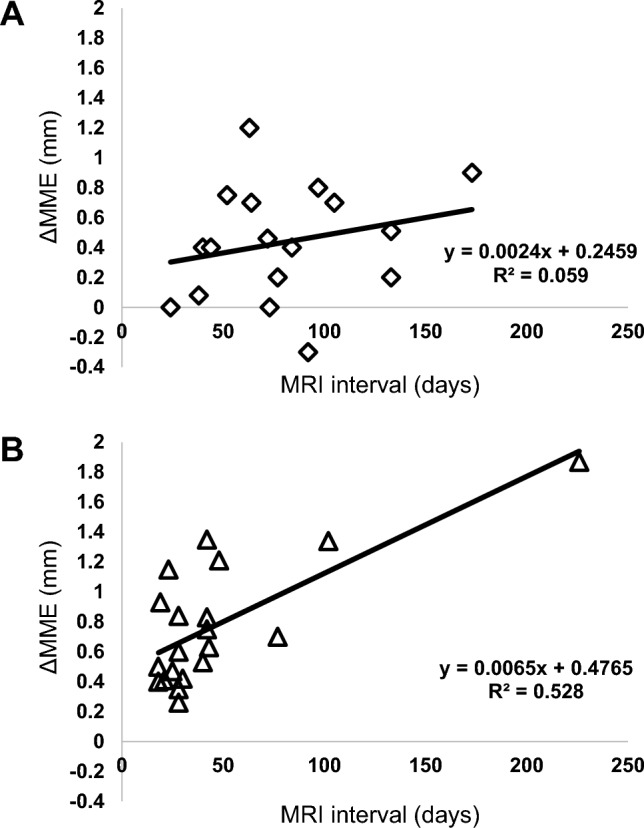
Scatter plot of the correlation between ΔMME and MRI interval. **a** Patients with nonoperative management (R^2^ = 0.059). **b** Patients with pull-out repair (R^2^ = 0.528). *MRI* magnetic resonance imaging, *ΔMME* the increase in medial meniscus extrusion

Linear regression analysis revealed a weak correlation between MRI interval and ΔMME in the nonoperative management group (ΔMME = 0.0024 × MRI interval + 0.2459, R^2^ = 0.059) (Fig. 3a) but a moderate correlation in the pull-out repair group (ΔMME = 0.0065 × MRI interval + 0.4765, R^2^ = 0.528) (Fig. 3b).

**Fig. 4:**
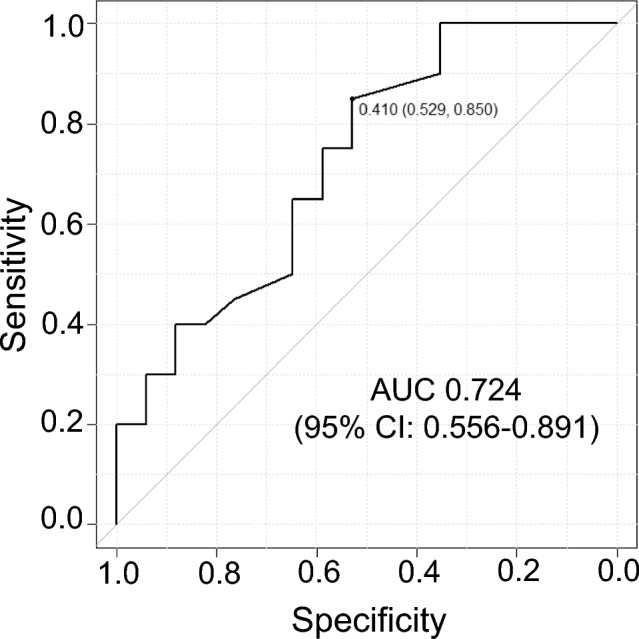
Cut-off value of ΔMME for partial MMPRTs requiring surgical intervention. The cut-off value of ΔMME for partial MMPRTs requiring surgical intervention was 0.41 mm, with a sensitivity and specificity of 85.0% and 52.9%, respectively. *MMPRTs* medial meniscus posterior root tears. *ΔMME* the increase in medial meniscus extrusion, *AUC* area under the curve, *CI* confidence interval

In the ROC construction, the cut-off value for ΔMME requiring surgical intervention was 0.41 mm, with a sensitivity and specificity of 85.0% and 52.9%, respectively (Fig. 4).

The ICC for the MME measurements was 0.965 and 0.957 for intra- and inter-observer repeatability, respectively.

A post-hoc power analysis for identifying the differences in ΔMME between the nonoperative and pull-out repair groups showed 72.8% power and a critical I-value of 0.05 (G*Power, version 3.1.9.7).

## Discussion

This study’s key findings indicated that the pull-out repair group had greater MME progression in a shorter time than the nonoperative management group. Additionally, patients with partial MMPRTs requiring surgical treatment showed a time-dependent increase in MME.

Most reports regarding conservative treatment have focused on complete MMPRTs. The 5-year follow-up of conservative treatment of MMPRTs has been reported to be poor with respect to arthropathic changes and clinical scores [10]. The large meniscus extrusion ratio has been reported as the most reliable poor prognostic factor for the conservative treatment of MMPRTs [11]. Kim et al. has also reported that age and MME of > 63.5 years and > 3 mm, respectively, are poor prognostic factors [12]. With conservative treatment, MMPRTs may not restore the original anatomic structure and function and may induce joint space narrowing and arthritic changes over time.

Only a few reports have summarized conservative treatment outcomes for partial MMPRTs. Therefore, the conservative treatment outcomes of partial MMPRTs are unclear, and the indications for surgery and the appropriate timing of surgery remain controversial. A report focusing on MME regarding surgical indications for partial MMPRTs by Furumatsu et al. found that large MME (particularly > 3 mm) in patients with partial MMPRTs was indicative of surgery [13]. No significant difference was found between the two groups of patients with MME > 3 mm on the first MRI scan in this study. However, the number of patients with MME > 3 mm at the second MRI scan was significantly greater in the pull-out repair group than in the nonoperative management group. Furthermore, MME > 3 mm on the second MRI scan frequently required a pull-out repair (odds ratio, 4.286; 95% confidence interval, 1.058–17.363). Additionally, a ΔMME was associated with the need for surgery.

The MME progression suggests hoop function disruption and the need for meniscal repair to re-establish meniscus function. MME is also associated with joint space narrowing and osteoarthritis progression [14]. MME in asymptomatic adults was previously reported to be 1.64 mm [15], progressing shortly after the MMPRTs onset [16]. It has also been reported that complete MMPRTs showed MME progression of 1.1 mm over a 47.8-day average and approximately 0.02 mm per day correlation between MRI interval and ΔMME [17]. In this study, patients with partial MMPRTs who required surgical treatment had a time-dependent increase in MME of 0.0065 mm per day. The progression of MME with partial MMPRTs requiring surgical intervention was slower than that with complete MMPRTs. However, due to the time-dependent increase in MME, meniscal repair must be performed without delay when necessary. Therefore, MRI rechecks at regular intervals to assess the extent of MME progression may aid decision-making in terms of surgical intervention without any delay. Furthermore, this study’s findings showed that ΔMME ≥ 0.41 mm on MRI retests at approximately 1–2 months intervals may help to determine surgical intervention.

The protocol followed for conservative treatment of MMPRTs is an important aspect of successful conservative treatment. The protocol for complete MMPRTs is relatively uniform and includes 12 weeks of physical therapy and pain control with non-steroidal anti-inflammatory drugs [18], 12 weeks of prohibited squats and stairs, pain control, and weight loss instructions [11]. A report established that 12 weeks of rehabilitation resulted in symptom and function improvement, whereas osteoarthritis progressed [19]. Our conservative treatment protocol for partial MMPRTs was similar in duration. Additionally, deep flexion of the knee joint was prohibited. Therefore, appropriate conservative treatment for partial MMPRTs should also be considered in the future.

This study has some limitations. First, it was a retrospective study. Second, the sample size was small. Third, patients who underwent nonoperative management may include those requiring future surgical interventions. Therefore, further studies are needed with larger sample sizes to determine the long-term course.

In summary, patients with partial MMPRTs who required surgical treatment had greater MME progression in a shorter time and a time-dependent increase in MME. Therefore, in cases with strong or persistent symptoms, even in patients with partial MMPRTs, MRI retests should be performed approximately 1–2 months apart, and a ΔMME ≥ 0.41 mm may indicate surgical intervention.

## Data Availability

The datasets generated and analysed during the current study are available from the corresponding author upon reasonable request.
